# Defining Higher-Risk Chronic Myeloid Leukemia: Risk Scores, Genomic Landscape, and Prognostication

**DOI:** 10.1007/s11899-022-00668-2

**Published:** 2022-08-06

**Authors:** Nur Hezrin Shahrin, Carol Wadham, Susan Branford

**Affiliations:** 1 Department of Genetics and Molecular Pathology, Centre for Cancer Biology, SA Pathology, Adelaide, South Australia 5000 Australia; 2 School of Pharmacy and Medical Science, Division of Health Sciences, University of South Australia, Adelaide, Australia; 3 School of Medicine, Faculty of Health and Medical Sciences, University of Adelaide, Adelaide, Australia

**Keywords:** Chronic myeloid leukemia, Risk scores, Prognostication, Comorbidities, Genomic landscape

## Abstract

**Purpose of Review:**

The chronic myeloid leukemia (CML) treatment success story is incomplete as some patients still fail therapy, leading to end-stage disease and death. Here we discuss recent research into CML incidence, the role of comorbidities on survival and detecting patients at risk of failing therapy.

**Recent Findings:**

The incidence of CML has fallen markedly in high social-demographic index (SDI) regions of the world but there is disturbing evidence that this is not the case in low and low-middle SDI countries. Now that CML patients more frequently die from their co-morbid conditions than from CML the Adult Comorbidity Evaluation-27 score can assist in risk assessment at diagnosis. Non-adherence to therapy contributes greatly to treatment failure. A good doctor-patient relationship and social support promote good adherence, but patient age, gender, and financial burden have negative effects, suggesting avenues for intervention. Mutations in cancer-associated genes adversely affect outcome and their detection at diagnosis may guide therapeutic choice and offer non-BCR::ABL1 targeted therapies. A differential gene expression signature to assist risk detection is a highly sought-after diagnostic tool being actively researched on several fronts.

**Summary:**

Detecting patients at risk of failing therapy is being assisted by recent technological advances enabling highly sensitive genomic and expression analysis of insensitive cells. However, patient lifestyle, adherence to therapy, and comorbidities are critical risk factors that need to be addressed by interventions such as social and financial support.

## Introduction

Chronic myeloid leukemia (CML) is a hematological malignancy primarily affecting older adults. The initiating event is a reciprocal fusion of chromosomes 9 and 22 in hematopoietic stem cells [1]. This generates a chimeric Philadelphia chromosome in which the BCR::ABL1 fusion protein is constitutively activated, a prerequisite for the pathogenesis of CML. Patients are usually diagnosed in initial indolent chronic phase (CP), which can often be asymptomatic so is commonly detected by routine blood tests. Therefore, people who have reduced access to basic medical care or those who do not normally require regular medical check-ups risk later detection. If left untreated the disease inevitably progresses to an accelerated phase (AP), blast phase (BP), and death.

Since the introduction of imatinib, the first BCR::ABL1 targeted tyrosine kinase inhibitor (TKI), more than twenty years ago, CML has transformed from a lethal illness to a chronic disease. Progression to AP or BP has reduced from 1.5–3.7% per year to between 0.3 and 2.2% per year [2]. However, there remain a minority of patients who fail to meet treatment milestones, and progress to AP/BP when TKIs are no longer effective. At BP, which can appear as a myeloid or lymphoid blast phenotype, the only curative option is allogeneic stem cell transplantation, but this carries risks of increased morbidity and mortality [3]. The more potent second generation TKIs (2GTKI) dasatinib, nilotinib, and bosutinib induce more rapid and profound molecular responses than imatinib, which may reduce the likelihood of progression [4–6]. The major mechanism of TKI resistance is point mutations in the BCR::ABL1 tyrosine kinase domain (TKD), detectable in approximately 30% of resistant CP patients and ~ 60% of patients in AP/BP [7].

Ideally, patients at high risk of treatment failure would be identified at diagnosis, enabling early treatment optimization. However, there is no recommended diagnostic tool that will accurately predict treatment response or inform specific therapy choice. Treatment intervention during therapy is based on molecular and cytogenetic findings. Patient lifestyle and adherence to medication also contribute to disease incidence and response to treatment. In this review, we discuss the most recent research into identifying patients at risk of treatment failure and progression.

## Clinical Risk Assessments

The Sokal risk score was developed in 1984 during the pre-TKI era when patients were treated with cytoreductive chemotherapies busulfan and later hydroxyurea [8]. Older people had a poorer response to these treatments and age was a factor in the score algorithm, which also included weighted scores for spleen size, and peripheral blood platelet and blast counts. The Hasford score published in 1998 predicted risk for patients treated with interferon alpha, adding eosinophil and basophil counts to the variables associated with risk [9]. Both Sokal and Hasford scores assigned patients to low, medium, or high risk.

Age does not reduce imatinib responsiveness; therefore, the Sokal score overestimated risk in older patients. The European Treatment and Outcome Study (EUTOS) [9] score was developed in 2011 to predict response following 18 months of treatment. The EUTOS score, based on spleen size and basophil count, predicts risk more accurately than the Sokal score for patients treated with TKIs. Such has been the success of TKIs, most patients now die from other causes. To account for this, the EUTOS long term survival score (ELTS) was developed to predict death from CML, defined as death occurring after recorded progression to AP or BP [10]. The ELTS better predicts long term survival than the Sokal score [11].

Clinical risk scores are recommended to be determined prior to commencing TKI therapy but are not used to guide treatment decisions. The 2GTKIs will achieve a more rapid and deeper molecular response than imatinib, reducing the likelihood of progression in an intermediate- or high-risk patient. The National Comprehensive Cancer Network (NCCN) advises using Sokal, Hasford, or ELTS score to determine risk, whereas the European LeukemiaNet (ELN) recommends using only ELTS due to the potential for inappropriate treatment arising from incorrect stratification [7, 12]. The NCCN suggest that 2GTKIs may be preferred for patients with intermediate or high-risk [12]. However, the greater weight assigned to older age by Sokal places more patients in higher risk categories and could prioritize 2GTKI in preference to imatinib. As older patients are more likely to have comorbidities, including cardiovascular disease, this can lead to detrimental effects as discussed in the next section. The ELN assign high risk ELTS score as a warning at baseline but make no recommendation on the selection of TKIs for these patients based on the clinical risk score [7].

## Prevalence of Comorbidities in CML Patients and Impact on Outcome

**Fig. 1:**
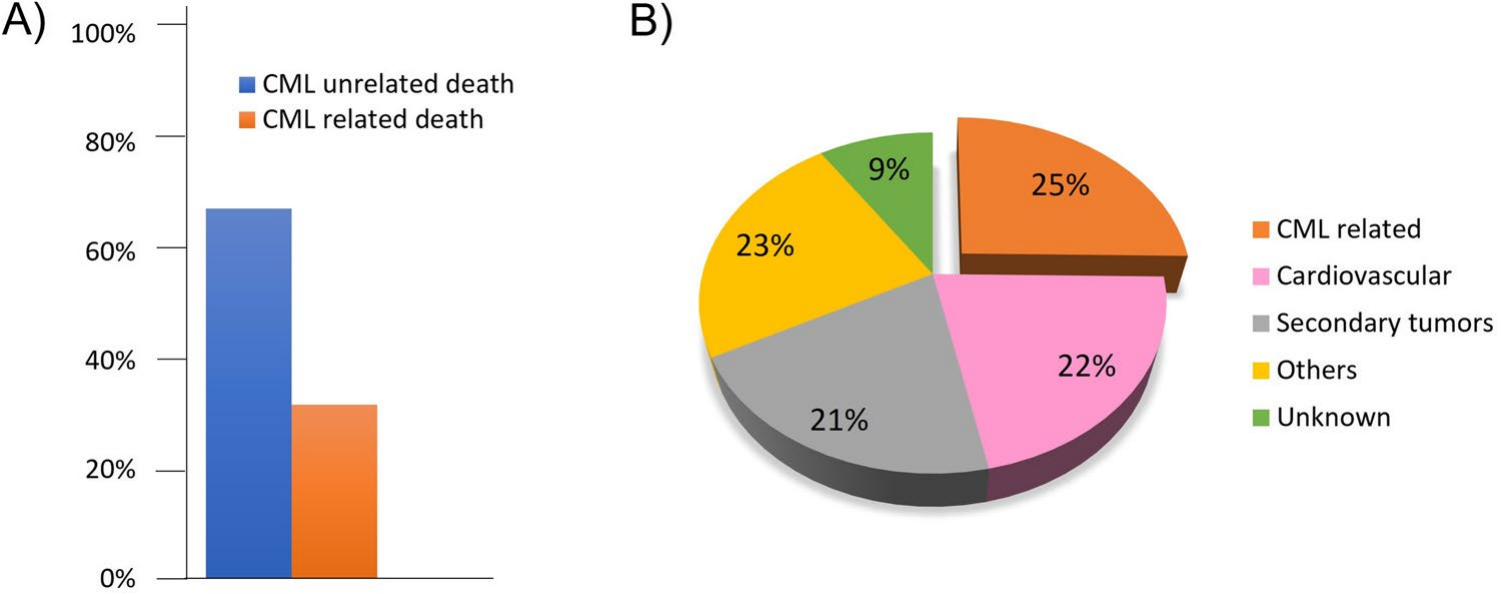
Impact of comorbidities on the overall survival of CML patients. The causes of death as reported in references 14–19 were examined. These studies included patients treated with various TKIs. **A** The bar chart shows that the cause of death in CML patients in these studies was mainly associated with reasons other than CML. **B** The pie chart illustrates the breakdown of the CML-unrelated causes of death. CML-related death, which resulted from CML progression or due to transplant complications, accounted for a quarter of the causes of death. Other causes of death included sepsis, terminal kidney insufficiency, cerebral bleeding, and pneumonia. Cardiovascular diseases encompassed cardiac insufficiency, myocardial infarction, cerebral stroke, and cardiac arrhythmias. Secondary tumors comprised prostate, colon, breast, lung, and bladder cancers

There is no doubt that treatment with TKIs and molecular monitoring has resulted in good overall survival of CML patients, where the life-expectancy approaches that of the general population [13]. However, this is not the case when CML patients have comorbidities, in which patients are much more likely to die from their comorbid conditions than from CML (Fig. 1) [14–20]. Patients with CML often have another medical condition at a higher incidence than in the general population. Some studies have reported that nearly 50% of CML patients had at least one comorbid condition [21–23]. Prominently, the overall survival was significantly lower for those with comorbidities compared to those without comorbidities at the time of their leukemia diagnosis, as reported in a randomized trial of 1519 patients in the CML study IV [15]. Comorbidities are a factor when considering the choice of TKI for newly diagnosed patients [7]. For example, the risk of cardiovascular events over 10 years for patients treated with frontline nilotinib is approximately 4-times higher than those treated with frontline imatinib [5]. Certain comorbidities that exacerbate the risk of cardiovascular events are a contraindication for using nilotinib as frontline therapy.

In 1987, the Charlson comorbidity index (CCI) was introduced to predict death for patients with specific comorbid conditions [24]. The CCI scoring system was later refined to include the prediction of overall survival for cancer patients with comorbidities [25]. In CML, Uemura et al. implemented a CCI scoring system in their study involving 79 CML patients categorized into a CCI score of 2 to 11 at diagnosis [16]. They showed that patients with a CCI score of > 3 had significantly shorter survival after diagnosis than the cases that scored < 2 points. Another study in Germany reported that 54 of 260 CML patients died during follow-up [14]. More patients died from comorbidities than from CML and death was strongly associated with the CCI index at diagnosis. Of the 54 patients, 20 (37%) died due to comorbidities, while 13 (26%) died due to CML. Survival was the poorest for patients with a CCI of ≥ 7 [14].

The prediction of outcomes based on comorbidities at diagnosis may be enhanced by combining the Adult Comorbidity Evaluation-27 score (ACE-27) with CCI scores [26]. ACE-27 is an index that assesses the burden of comorbidities. A recent study of 524 patients reported that ACE-27 score predicted outcome, including overall survival and event-free survival in CP-CML patients [27]. Interestingly, patients with a higher ACE-27 score were less likely to achieve a complete cytogenetic response and molecular responses.

Comorbidities at the time of diagnosis are among the most important predictors of long-term survival for CML patients. Therefore, CCI and ACE-27 scoring (Table 1) could be included as a prognostic instrument in defining higher risk CML patients as these might help treatment compliance and longevity. Patients with high CCI and ACE-27 will need close monitoring for signs of the development of adverse drug reactions during TKI treatment.

**Table 1:** Charlson comorbidity index (CCI) is scored based on the comorbid conditions. The scoring is a scale from 0 to 6 points depending on the item’s strength and connotation with 1-year mortality (https://www.mdcalc.com/charlson-comorbidity-index-cci). The Adult Comorbidity Evaluation-27 (ACE-27) is a validated comorbidity index developed through modifications and additions of the Kaplan-Feinstein comorbidity index. The scoring point is 0–3 based on the severity of 27 conditions (https://m.medicalalgorithms.com/adult-comorbidity-evaluation-27-ace-27)

CCI score	Conditions
Score 1	Myocardial infarct, congestive heart failure, peripheral vascular disease, cerebrovascular accident or transient ischemic attack, dementia, chronic obstructive pulmonary disease, connective tissue disease, peptic ulcer disease, mild liver disease, diabetes and age 50–59 years
Score 2	Hemiplegia, moderate or several renal diseases, diabetes with end organ damage, any tumor without metastases, leukemia, lymphoma, and age 60–69 years
Score 3	Moderate or severe liver disease and age 70–79 years
Score 4	≥ 80 years
Score 6	Metastatic solid tumor, AIDS
ACE-27 score	Criteria
None, 0	• No comorbidity
Mild, 1	• Comorbid conditions that are manageable with or without medications but do not require hospitalization • Not limiting the daily routine
Moderate, 2	• Comorbidities that require active treatment modifications or surgery • Residual disability affecting daily routine
Severe, 3	• Comorbidities that cause major complications or irreversible end-organ damage • Disability resulting in full support for daily routine
27 Conditions Myocardial infarct, coronary artery disease, congestive heart failure, arrhythmias, hypertension, venous disease, peripheral arterial disease, respiratory system, hepatic disease, stomach or intestinal disease, pancreatic disease, end-stage renal disease, diabetes mellitus, stroke, dementia, paralysis, neuromuscular, rheumatologic system, AIDS, solid tumors, leukemia and myeloma, lymphoma, alcohol, illicit drugs, obesity	

## Biomarkers of TKI Resistance and Progression

Untreated leukemic multipotent-progenitor cells are highly proliferative and genetically unstable, leading to the accumulation of additional chromosomal abnormalities, clonal evolution, and disease progression. Thus, the time from diagnosis to initiating treatment is critical to achieving a good response. As the early stages of the disease are often asymptomatic, patients are diagnosed at varying time points from the initiating fusion event. Accordingly, the period from disease onset to initiating treatment may vary widely, contributing to the heterogeneity of the molecular background of CML in chronic phase patients. There is much active research into this diversity with the aim of identifying biomarkers of risk. Potential biomarkers include additional chromosome abnormalities (ACAs), somatic variants in cancer genes, abnormal gene expression, additional fusions, and gene deletion.

### Additional Chromosome Abnormalities

Constitutive BCR::ABL1 kinase activation induces genomic instability, potentially leading to the acquisition of ACAs, which may alter risk depending on the timing and type of ACA. The ELN recommendations identify high risk ACAs to include + 8, a second Ph-chromosome (+ Ph), i(17q), + 19, − 7/7q-, 11q23, or 3q26.2 aberrations, and complex aberrant karyotypes [7]. High risk ACAs predict a poorer response to TKIs and a higher risk of progression. The ELN categorizes ACAs detected at any time while on treatment as TKI failure.

Not all ACAs acquired during therapy carry the same risk. Thirty percent (183/610) of a cohort of 2015 CML patients were found to have trisomy 8, with or without other ACAs [28]. Whereas trisomy 8 as the sole ACA predicted a good response to TKIs and good overall survival, in the presence of other ACAs, trisomy 8 patients had a poor response to TKIs and reduced OS. Interestingly, a retrospective review of 1,510 imatinib treated, CML IV study patients found that in patients with blast counts as low as 1%, who would otherwise be classified as CP, detection of high-risk ACAs predicted TKI resistance and death prior to expansion of the leukemic clone [29•].

The emergence of ACAs during TKI treatment increases risk, however, the relevance of ACAs at CP diagnosis has been controversial. In a study of 603 patients treated with TKIs and followed up for 5 years, ACAs detected at diagnosis (29/603) did not confer worse prognosis. However, the authors note the absence of high risk ACAs i(17)q or 3q26 [30], which may have influenced the outcome [31]. Nevertheless, diagnostic cytogenetic data collected for 763 of 812 subjects on the SPIRIT2 trial, comparing front-line dasatinib with imatinib, showed that 35.7% (5/14) with ACAs progressed, compared to 2.3% (16/736) of patients with only the Ph chromosome [32]. The authors concluded that ACAs at diagnosis predict disease progression independently of Sokal or ELTS scores. However, there were only 6 patients in this large study with one or more high risk ACAs who progressed. A meta-analysis of the data is required to clarify this question as a sufficiently large trial is unlikely to be feasible. The ELN recommends that detection of a high-risk ACA at diagnosis should be classified as a warning [7].

Between 2 and 10% of patients treated with TKIs have been reported to have ACAs in Ph-negative (Ph-) cells, with unknown relevance. A study in which 58/598 CP subjects had an ACA in Ph- cells found that ACA/Ph-, except for Y chromosome deletion, independently increased the risk of progression [33]. Moreover, a large, retrospective multicenter study of ACA/Ph- CML patients with a prolonged follow up (median 6.47 years) found that patients with -7/del(7q) (26/102 patients) more frequently had signs of dysplasia, with poor event-free and progression-free survival [34]. However, ACAs in Ph- cells have no adverse impact on overall prognosis according to ELN recommendations and NCCN guidelines.

### BCR::ABL1 Tyrosine Kinase Domain Mutations

Imatinib and subsequent generations of TKI bind to the kinase domain (KD) of BCR::ABL1, with the exception of asciminib, leading to the clonal expansion of KD-mutated TKI resistant cells. Sanger sequencing can only detect mutations with a variant allele frequency (VAF) of ~ 10–15%. Mutation detection at lower VAF would reduce risk of disease progression by changing treatments. We and others have demonstrated the importance of low-level *BCR::ABL1* mutations and TKI resistance using mass spectrometry [35–37].

Kizilors et al. screened for low-level *BCR::ABL1* mutations in consecutive newly diagnosed CML patients, including both optimal responders and those resistant to TKIs [38]. They detected *BCR::ABL1* mutations in 25/121 (21%) patients. Four patients with a mutation detected after 3 months of TKI treatment all progressed to AP. Low-frequency mutations later became the dominant clone when treatment was unchanged and predicted poor outcomes at 5 years [38]. A prospective multicenter study of 236 consecutive patients with warning/TKI failure detected low level mutations (VAF of 3 to 20%) in 34% of patients and therapy change was indicated in half of these [39]. Notably, clonal selection occurred in all 16 cases with a low-level mutation known to be resistant to the existing TKI, leading to TKI failure after 3 to 12 months.

In summary, low VAF *BCR::ABL1* mutations predict clonal selection and disease progression. Accordingly, the ELN recommend using NGS to detect *BCR::ABL1* mutations [7].

### Cancer-Related Gene Mutations

Evidence has accumulated that somatic mutations in cancer-related genes accumulate during progression from CP to AP/BP [40–43, 44•, 45•]. There are several genes recurrently mutated at AP/BP, including *ASXL1*, *RUNX1*, and *IKZF1*. In a study enriched for TKI non-responders, mutations were detected in 30% of CML patients in CP (*n* = 90), and 11/20 patients who progressed to BP had somatic variants at CP [41]. Imatinib but not 2GTKI-treated patients with somatic variants had poorer outcomes compared with patients without variants at diagnosis.

Mutational load increases with progression from CP to AP/BP [43]. Acquiring new mutations following commencement of treatment predicts treatment failure; however, clearance of mutations found at CP is not predictive of outcome [46]. In our study of poor versus optimal responders, 10/16 patients (62%) with *BCR::ABL1* mutations at BP who were sequenced at prior time points, had cancer gene variants that predated the *BCR::ABL1* mutations [40], suggesting that cancer gene variants may predispose the leukemic clone to acquire *BCR::ABL1* mutations.

Integrated analysis of multiple forms of data enables a deeper understanding of the somatic changes in CML progression. In 2020, Ko et al. employed multi-omic analysis to interrogate the genome, transcriptome and epigenome of matched CP and BP pairs [44•]. It was found that more genes were affected by copy number alterations (CNAs) in BP samples than single nucleotide variants (SNVs) or small insertions or deletions (indels). The gene most impacted by CNAs was *IKZF1*, and deletions commonly occurred in chromosomes 7, 9, and 14 [44•]. We also found frequent deletions in these regions at BP [40, 47] in addition to revealing gene fusions as frequent events driving disease progression. Using a combined sequencing strategy of whole exome and RNA sequencing, we found mutated cancer genes in all 39 patients in BP, including SNVs/indels, focal deletions, and gene fusions [40, 47].

A study by Ochi et al., of 52 CP-BP matched pairs showed that TKI treatment suppresses non-*BCR::ABL1* mutation acquisition [45•]. With an expanded number of samples, the authors found that 126/136 BP samples had at least one mutation or CNA and that *ASXL1* mutations, complex CNAs (defined as ≥ 3 CNAs), i(17q) and + 21 were independent predictors of poor prognosis in TKI treated patients.

As discussed previously in more detail [42], the body of evidence implicating somatic mutations in CML disease progression is now considerable. Screening for mutations in resistant patient samples will potentially assist future risk assessment and identify alternative non-BCR::ABL1 treatment alternatives [7].

### Mutations that Pre-exist the Acquisition of BCR::ABL1

Longitudinal studies of CML patients consistently reported the emergence of BCR::ABL1 in a pre-existing clonal population carrying a clonal hematopoiesis-related mutant, or the expansion of BCR::ABL1-negative clones during therapy [40, 41, 46, 48]. Clonal hematopoiesis is an age-related abnormal expansion of cells carrying a somatic mutation that confers a growth advantage [49]. The mutated genes are those associated with blood cancer and the most frequently mutated genes are *DNMT3A*, *TET2*, and *ASXL1*. These genes are also among those reported in patients with CML [42]. Clonal hematopoiesis clones give rise to mutated immune effector cells with a proinflammatory profile that exacerbate diseases with a chronic inflammatory component, such as cardiovascular disease, and are associated with all-cause mortality [50]. The risk of cardiovascular disease associated with clonal hematopoiesis is substantial and is as great or greater than common risk factors [49].

Mutations that pre-exist the acquisition of BCR::ABL1 can persist and expand in patients successfully treated with TKI therapy [46]. These expanded mutant clones contribute to clonal hematopoiesis in CML patients in remission, which has also been described for patients with acute myeloid leukemia (AML) [51]. Mutations in *DNMT3A*, *TET2*, and *ASXL1* persisted in AML patients in remission and were not associated with relapse. Similarly, in CML, the persistence of clonal hematopoiesis-related mutations in remission has not been associated with TKI resistance [46]. The impact of cancer-related gene mutations detected at the time of CML diagnosis may vary depending on whether they were acquired before or after *BCR::ABL1*. Acquisition of mutations after *BCR::ABL1* may signify a more genomically unstable disease, prone to acquisition of potentially damaging additional mutations. These may be a marker of high risk of treatment failure. Larger studies are required to determine the frequency of clonal hematopoiesis in CML and to unravel the significance of mutants that pre-exist the acquisition of BCR::ABL1.

Given the association of more potent TKIs with comorbidities, such as cardiovascular disease [5], an important question for CML patients that remains unanswered is whether clonal hematopoiesis could play a role in the exacerbation or development of comorbidities during TKI therapy. This question is particularly relevant since most CML patients will receive life-long TKI therapy, where life-expectancy approaches that of the general population. Interestingly, a study that assessed factors associated with arterial occlusive disease for nilotinib-treated CML patients reported a significantly higher frequency of clonal hematopoiesis mutants in patients with arterial occlusive disease compared to those without [52].

The NCCN suggest that a myeloid mutation panel be considered for patients with accelerated or blast phase to identify BCR::ABL1-independent resistance mutations [12]. However, the NCCN or the ELN do not yet provide guidance on how to assess risk and/or identify alternative treatment based on these emerging data. Currently, testing for cancer-associated mutations in CML is largely undertaken in the research setting. Therefore, there is still much to be learned on the role of these mutations for resistance. Expanded studies will provide evidence to inform clinical practice guidelines.

### Altered Gene Expression and Epigenetic Changes

The transition from CP to AP/BP is marked by changes in gene expression, a promising biomarker of progression. Many attempts have been made over the last decade to derive biomarkers of disease progression from gene expression data, propelled by the rapid technological advances over the same period (reviewed in [53]). To date, there is no diagnostic gene expression panel for CML but progress towards this goal continues.

In a microarray analysis of CP, AP, and BP, Radich et al. reported significant changes in expression of 3000 genes [54]. The data suggested a two-step path to progression rather than three-step. Computational analysis of these data subsequently found characteristic gene expression differences between all three CML phases and identified 24 genes with high connectivity as potential major regulators of progression. Some have previously been associated with CML or other leukemias [55].

Analysis of RNA sequencing differential gene expression data of optimal- versus poor-responders to imatinib [40] disclosed an upregulation of genes involved in V(D)J recombination, including *RAG1*/*2* and *DNTT* at lymphoid BP [47]. All patients with elevated *DNTT* at CP diagnosis progressed to lymphoid BP by 12 months [47], consistent with RAG-mediated recombination induced deletions and fusions driving progression to lymphoid BP.

An integrated multi-omics approach to elucidate the molecular events leading to BP found recurrent mutations in the polycomb repressive complex 1 (PRC1) and PRC2 pathways [44•]. The PRCs are multiprotein complexes that catalyze histone modifications, inducing changes to the transcription machinery culminating in gene repression [56]. By integrating mutation, transcription, and epigenetic datasets. Ko et al. concluded that BP progenitors undergo PRC driven epigenetic reprogramming [44•]. In summary, BP-specific hypermethylation by PRC2 enzyme EZH2 inhibits cell differentiation, whereas the PRC1 catalytic protein BMI1 inhibits cell death [44•].

Recent additions to the differentially expressed genes which may serve as potential biomarkers of progression or TKI resistance include downregulation of the large HECT E3 ubiquitin ligase HERC1 in leukemic cells [57], FAM167A induced activation of the noncanonical NF-κB pathway in TKI-resistant CML cells [58], and upregulation of exosomal proteins RPL13 and RPL14 in the plasma of imatinib resistant patients [59]. Additionally, the MS4A3 transmembrane protein is downregulated in CML progenitor cells preventing differentiation [60]. Quiescent stem cells are resistant to TKIs and thereby pose an ongoing risk of disease progression and transformation to BP. Targeted delivery of MS4A3 may be a means of inducing differentiation and thus elimination of quiescent stem cells.

## Risks of Non-adherence

Non-adherence to therapy is a key treatment-failure risk and a challenge for treating clinicians [61–66]. Anything less than complete adherence in patients treated with imatinib is associated with increased risk of a poor response [65]. Although TKIs have been lifesaving, chronic illness and lifelong therapy are financial and quality of life burdens affecting many aspects of patients’ lives. Younger female patients are unable to start a family while on TKIs, off-target effects can reduce wellbeing and requirements for fasting before and after some 2GTKIs adversely affect social and family life [67]. Regular quantitative PCR monitoring of *BCR*::*ABL1* transcripts increases adherence and reduces risk of progression [64], but non-adherence is still an unresolved problem.

A small study into beliefs and obstacles associated with adherence found that although most considered their adherence to be good to excellent, 18% missed at least one dose in the preceding seven days [68]. A systematic review of nine studies conducted in the USA (*n* = 3), Europe (*n* = 3), and Asia (*n* = 3) found that complex interventions, such as education and encouragement delivered by healthcare professionals, could improve adherence to TKI therapy. However, the effect in some of the studies was small or none. Furthermore, only one study showed a significant association between intervention and clinical outcomes [66].

An early study monitored drug adherence for 3 months using a microelectronic monitoring system to record each time the medication bottle was opened [69]. Adherence of ≤ 90% was reported in 26% of patients and was associated with significantly inferior response. Younger patients and those who increased the dose of imatinib were less adherent. A recent USA study aimed to classify adherence patterns to understand the factors that influence adherence [63]. Four groups were identified among 2049 people: stable adherent; never adherent; initially non-adherent becoming adherent; or initially adherent becoming non-adherent. Older age and taking additional medications were factors associated with stable adherence. Notably, women were less likely to be in the stable adherent group. There was an overall trend for long-term adherence to be lower than initial adherence. At 12 months, 22% of individuals were taking their medication less than 80% of the time [63].

A retrospective study of 2870 adult CML patients in Korea reported very high levels of adherence and that adherence level affected outcomes [70]. Adherence was measured by the medication possession ratio (MPR) that is the number of pills available divided by the number of days. The median MPR was reported as 0.99. However, lower adherence and overall survival were reported for women, those with lower health insurance and people aged over 70 years. Interestingly, the USA study [61] found a relationship between financial burden and adherence over time. The US lacks a universal health care scheme and the percentage of population in poverty is much higher than in the Organisation for Economic Co-operation and Development (OECD) countries (17% vs 9%) [71]. However, a compulsory health insurance scheme covers the entire Korean population (https://www.nhis.or.kr/static/html/wbd/g/a/wbdga0301.html). The Korean study also showed increased adherence among people with higher health insurance cover, but universal insurance mitigates the financial burden for all.

A European study of 2546 CML patients from 63 countries found only 32.7% of participants were highly adherent [72]. The reasons for non-adherence were complex but like the previously discussed studies, men were more adherent than women. Single dose medication, a good doctor patient relationship and not living alone also correlated with higher adherence. No payment was required for 80% of respondents, but of the remaining 20%, personal co-payment of more than 50 Euros per month negatively affected adherence (*p* = 0.0088).

Some of the variability between studies may be due in part to differences in ways of assessing non-adherence. Filling a prescription does not mean that all tablets are taken as prescribed, and self-reported non-adherence is likely to be under-reported. However, there are some consistent findings across studies such as financial disincentives and lower adherence in women that suggest outcomes could be improved if these are addressed.

## Conclusion

Maintaining patients in chronic phase CML is essential to avoid CML-induced death, yet we lack accurate predictive tools to inform therapeutic choices. The emerging role of genomic abnormalities in disease progression and for predicting response to TKI therapy offer hope of enhanced risk prediction and the identification of potential targets for therapy. However, leukemia-related biological factors are not the only ones influencing risk for CML patients where comorbidities and adherence to therapy also influence response and outcome.
